# Diphoton resonance in F-theory inspired flipped $\mathrm{SO}(10)$

**DOI:** 10.1140/epjc/s10052-016-4432-y

**Published:** 2016-10-24

**Authors:** George K. Leontaris, Qaisar Shafi

**Affiliations:** 1 Physics Department, Theory Division, Ioannina University, 45110 Ioannina, Greece; 2 Department of Physics and Astronomy, Bartol Research Institute, University of Delaware, Newark, DE 19716 USA

## Abstract

Motivated by the di-photon excess at 750 GeV reported by the ATLAS and CMS experiments, we present an F-theory inspired flipped $\mathrm{SO}(10)$ model embedded in $\mathcal{E}_6$. The low energy spectrum includes the three MSSM chiral families, vector-like colour triplets, several pairs of charged $\mathrm{SU}(2)_L$ singlet fields $(E^c, \bar{E}^c)$, as well as MSSM singlets, one or more of which could contribute to the di-photon resonance. A total decay width in the multi-GeV range can arise from couplings involving the singlet and MSSM fields.

## Introduction

Recently, the LHC experiments ATLAS and CMS, have reported an excess of di-photon events at an invariant mass around 750 GeV from data at LHC run 2 with *pp* collisions at $\sqrt{s}= 13$ TeV [1, 2]. The absence of data on other channels such as *WW*, *ZZ* and $Z\gamma$ indicate that the interpretation of these events cannot be accommodated within the Standard Model (SM). It has been suggested that an interpretation requires new physics beyond the known SM context. In fact this effect hints to the existence of singlets and vector-like states in the low energy spectrum of the theory. The observed resonance could be explained by a SM scalar or pseudoscalar singlet state *X* [3–8] with mass $M_X\sim 750$ GeV.^1^ This state could be generated by the gluon–gluon fusion mechanism while subsequently it decays to two photons. Schematically, this is described as follows:

1 $$\begin{aligned} gg \rightarrow X\rightarrow \gamma \gamma . \end{aligned}$$

In a renormalisable theory, the production and decay in this process can be realised through loops involving appropriate vector-like states. Remarkably, a common phenomenon in string theory model building is the occurrence of new singlet fields and vector-like exotic states in the massless spectrum of the effective low energy models which can mediate such processes [9–26].^2^

F-theory models in particular offer a wide range of possibilities [28, 29]. Unlike other string constructions, they admit exceptional gauge symmetries such as $\mathcal{E}_8$ and its subgroups, which incorporate naturally the concept of gauge coupling unification. Besides, when most of the successful old GUT groups are realised in an F-theory background they naturally predict vector-like pairs of quarks and leptons in the light spectrum. In fact, when the GUT symmetry is $\mathrm{SO}(10)$ or higher the appearance of such states is unavoidable [28].

Inspired by the above facts, in this note we construct a flipped $\mathrm{SO}(10)$ model embedded in an F-theory motivated $\mathcal{E}_6$ unified gauge group [30–32]. We show that this construction includes singlets as well as vector-like states which come with the quantum numbers of SM particles capable of mediating processes such as the di-photon production. In addition, we find that other vector-like states with exotic quantum numbers emerge from the adjoint decomposition.

The study of this alternative embedding is well motivated in F-theory constructions where the GUT symmetry can be as large as $$E_8$$. Indeed, in the restricted case of minimal $$\mathrm{SU}(5)$$, there is a unique assignment of the hypercharge generator in this group. However, there are many possibilities with a larger GUT symmetry and includes additional *U*(1) factors. In the case of $$\mathrm{SO}(10)\rightarrow \mathrm{SU}(5)\times U(1)_{X}$$, with the standard hypercharge assignment the extra $$ U(1)_{X}$$ factor is treated as a spectator, but there is no compelling reason for this. Similarly, in the $$E_6$$ case, there are two additional *U*(1) factors that could contribute to the hypercharge. Thus, different embeddings lead to distinct phenomenological predictions. In this work we wish to consider an alternative embedding of the hypecharge generator and try to assess the model in terms of its low energy predictions.

In order to obtain chiral matter we will assume the existence of a suitable four-form flux. Of course, the flux depends on the choice of the four complex dimensional Calabi–Yau (CY) manifold and the geometric properties of the divisor supporting the specific singularity ($\mathcal{E}_6$ in the present case). For our present purposes, however, we will work in the spectral cover approach where the properties of our local construction can be adequately described in the infinitesimal vicinity of the GUT divisor, and therefore, we will rely on the assumption that such a manifold exists.

The layout of the present paper is as follows. In the next section we present an $\mathrm{SO}(10)$ flipped model embedded in the $\mathcal{E}_6$ gauge symmetry. We discuss the basic properties of its spectrum and the predicted exotics. In Sect. 3 we derive the superpotential of the effective model emerging under the action of a $Z_2$ monodromy. Next, in Sect. 4 we focus on the existence of exotic vector-like pairs and singlet field which are suitable to contribute to the di-photon emission in *pp* collisions. We present our conclusions in Sect. 5.

## Effective flipped models from $\mathcal{E}_6$

In F-theory the gauge symmetry of the effective theory is linked to the geometric singularity of the compactification manifold. In the elliptic fibration these singularities are described by the sequence of the subgroups of the exceptional group $\mathcal{E}_8$. In the present F-theory construction we will analyse an $\mathrm{SO}(10)\times U(1)$ gauge symmetry which admits a natural embedding in the exceptional group $\mathcal{E}_6$. Therefore, with respect to the $\mathcal{E}_8$ we have the following breaking pattern:

$$\begin{aligned} \mathcal{E}_8\supset \mathcal{E}_6\times \mathrm{SU}(3)_{\perp }\supset \mathrm{SO}(10)\times U(1)_{X'}\times \mathrm{SU}(3)_{\perp } \end{aligned}$$

where, in accordance to the standard terminology, the $\mathrm{SU}(3)_{\perp }$ factor is considered as the group ‘perpendicular’ to $\mathcal{E}_6$ GUT divisor. We will assume a semilocal approach where the $\mathcal{E}_6$ representations transform non-trivially under $\mathrm{SU}(3)_{\perp }$. The matter content arises from the decomposition of the $\mathcal{E}_8$ adjoint ($\mathcal{E}_8\supset \mathcal{E}_6\times \mathrm{SU}(3)_{\perp }$)

$$\begin{aligned} 248\rightarrow (78,1)+(1,8)+(27,3)+(\overline{27}, \overline{3}). \end{aligned}$$

In the spectral cover approach the $\mathcal{E}_6$ representations are distinguished by the ‘weights’ $t_{1,2,3}$ of the $\mathrm{SU}(3)_{\perp }$ Cartan subalgebra subject to $t_1+t_2+t_3=0$, while the $\mathrm{SU}(3)_{\perp }$ adjoint ‘decomposes’ into singlets $1_{t_i-t_j}\equiv \theta _{ij}$. We introduce the notation

$$\begin{aligned} (1,8)\rightarrow \theta _{ij},\;\ (27,3)\rightarrow 27_{t_i},\; (\overline{27},\overline{3})\rightarrow \overline{27}_{-t_i}, \end{aligned}$$

while the $\mathcal{E}_6$ adjoint $\mathbf{78}$ is an $\mathrm{SU}(3)_{\perp }$ singlet and therefore carries no $t_i$ index. Since we are interested in a flipped $\mathrm{SO}(10)$ model, in the subsequent analysis we choose to accommodate the ordinary fermionic states and Higgs in the ${27}_{t_i}$. We further assume that the symmetry breaks though a non-trivial abelian flux which, at the same time, determines the chirality of the complete $\mathcal{E}_6$ representations $27_{t_{1,2,3}}$.

We start with the derivation of the flipped $\mathrm{SO}(10)$ model^3^ in an F-theory inspired context. We will assume that the bulk gauge group is $\mathcal{E}_6$, which breaks to $\mathrm{SO}(10)$ by turning on a $U(1)_{X'}$ gauge field configuration, where the particular $U(1)_{X'}$ is embedded in $\mathcal{E}_6$. Under the decomposition $\mathcal{E}_6\supset \mathrm{SO}(10)\times U(1)_{X'}$, the relevant $\mathcal{E}_6$ representations decompose as follows:

2 $$\begin{aligned}&\mathcal{E}_6\supset \mathrm{SO}(10)\times U(1)_{X'}\nonumber \\&78\rightarrow \mathbf{45}_0+\mathbf{1}_0+\mathbf{16}_{-3}+\overline{\mathbf{16}}_3\end{aligned}$$

3 $$\begin{aligned}&27\rightarrow \mathbf{16}_1+\mathbf{10}_{-2}+\mathbf{1}_4\end{aligned}$$

4 $$\begin{aligned}&\overline{27} \rightarrow \overline{\mathbf{16}}_{-1}+\overline{\mathbf{10}}_{2}+\mathbf{1}_{-4} .\end{aligned}$$

In principle, there are $\mathrm{SO}(10)$ zero modes in the adjoint $\mathbf{16}_{-3}$ and $\overline{\mathbf{16}}_3$ as well as in $\mathbf{27}$, which might accommodate chiral matter provided that $n_{16}-n_{\overline{16}}\ne 0$. In the next step, we break the $\mathrm{SO}(10)$ symmetry down to $\mathrm{SU}(5)\times U(1)_X$ by turning on a flux along $U(1)_{X}$, so that at this stage the symmetry breaking chain is

5 $$\begin{aligned} \mathcal{E}_6 \supset \mathrm{SO}(10)\times U(1)_{X'} \supset [\mathrm{SU}(5)\times U(1)_X]\times U(1)_{X'}.\nonumber \\ \end{aligned}$$

If we denote with $X,X'$ the corresponding abelian charges, for the flipped $\mathrm{SO}(10)$ case we define the following combination:^4^

6 $$\begin{aligned} Z=-\frac{1}{4} \left( X+5 X'\right) . \end{aligned}$$

Under the above symmetry breaking, the $\mathrm{SO}(10)$ representations decompose to various $\mathrm{SU}(5)$ multiplets. With respect to $\mathcal{E}_6\rightarrow \mathrm{SU}(5)\times U(1)_Z$, these have the following ‘charge’ assignments:

7 $$\begin{aligned} 27\rightarrow & {} \{10_\mathbf{-1}+\overline{5}_\mathbf{-2}+1_\mathbf{0}\}+\{5_\mathbf{2}+\bar{5}_\mathbf{3}\}+\mathbf{1}_\mathbf{-5}\end{aligned}$$

8 $$\begin{aligned} 78\rightarrow & {} \{24_\mathbf{0}+10_\mathbf{-1}+\overline{10}_\mathbf{1}+1_\mathbf{0}\} \nonumber \\&+\,\{10_\mathbf{4}+\bar{5}_\mathbf{3}+1_\mathbf{5}\} + \{\overline{10}_\mathbf{-4}+ 5_\mathbf{-3}+1_\mathbf{-5}\}\nonumber \\&+\,\mathbf{1}_\mathbf{0}. \end{aligned}$$

At the final stage, we break $\mathrm{SU}(5)\rightarrow \mathrm{SU}(3)\times \mathrm{SU}(2)\times U(1)_y$, where the hypercharge is defined to be the linear combination of the three abelian factors $U(1)_{X'}, U(1)_X, U(1)_y$ given by

9 $$\begin{aligned} Y =-\frac{1}{5} \left( Z+\frac{y}{6}\right) . \end{aligned}$$

We will require that the SM fermions and Higgs doublets reside on matter curves $\Sigma _{27}$ formed at the intersections of the GUT surface with other 7-branes. Employing the above hypercharge definition, the embedding of the SM states in the 27-representation of $E_6$ is as follows:10The symbol $\phi$ stands for a $\mathrm{SU}(5)$ singlet field while for all other SM states, we use the standard notation. As can be seen, compared to the standard $\mathrm{SO}(10)$ embedding, here we obtain a ‘flipped’ picture of the 5-plet and singlet representations, i.e., $\bar{5}_h\leftrightarrow \bar{5}_f$ and $1_{e^c}\leftrightarrow 1_{\phi }$. More precisely, compared to flipped $\mathrm{SU}(5)$, this $U(1)_Z$ definition flips $\bar{5}_{-2}$ with $\bar{5}_{3}$ and $1_0$ with $1_{-5}$. The fermion component $\bar{f}=\bar{5}_{-3}$ in this case is part of the $\mathbf{10}$-plet ($\in \mathrm{SO}(10)$), and the Higgs $\bar{h}=\bar{5}_{-2}$ is part of $\mathbf{16}$. Furthermore, the $\mathrm{SU}(5)$ singlet $\phi$ is electrically neutral and the right-handed electron is found in the $\mathrm{SO}(10)$ singlet $\mathbf{1}_{4}$.

In addition to the SM fields residing in the $\Sigma _{27}$ matter curves, there is also bulk matter emerging from the docomposition of the $\mathbf{78}$ representation. Namely:11We have used the symbols $\chi _0, \Phi$ for the two neutral singlets, while for the remaining content arising from the decomposition of ${ 24}\in \mathrm{SU}(5)$, we have introduced the notation

12 $$\begin{aligned} G_0+T_0+S_0= & {} (8,1)_0+(1,3)_0+(1,1)_0\end{aligned}$$

13 $$\begin{aligned} Q+\bar{Q}= & {} (3,2)_{\frac{1}{6}}+(\bar{3},2)_{-\frac{1}{6}}\end{aligned}$$

14 $$\begin{aligned} Q'+\bar{Q}'= & {} (3,2)_{-\frac{5}{6}}+(\bar{3},2)_{\frac{5}{6}}. \end{aligned}$$

In the above, *Q* has the standard quark doublet quantum numbers and $\bar{Q}$ is its complex conjugate, while $Q'+\bar{Q}'$ have exotic charges.

**Table 1:** $\mathcal{E}_6$ matter curves, their defining equations, the homology classes and the multiplicities in terms of the flux restrictions

Matter	Equation	Homology	$\# 27_{t_i}-\# \overline{27}_{-t_i}$
$27_{t_{1}}$	$a_1$	$\eta - 2 c_1 - {\chi }$	$n_1=\mathcal{F}_{U(1)}\cdot (\eta -2c_1-\chi )$
$27_{t_{3}}$	$a_4$	$-c_1 + \chi$	$n_3=\mathcal{F}_{U(1)}\cdot (\chi -c_1)$

In the standard (non-flipped) $\mathrm{SU}(5)$ theory, the $Q'+\bar{Q}'$ exotics emerge from the decomposition of the 24-adjoint. Hence, we observe that the flipped case interchanges $Q'+\bar{Q}'$ exotics in the adjoint of the standard $\mathrm{SU}(5)$, with the ordinary $Q+\bar{Q}$ within the $\mathbf{16}_{-3}+\overline{\mathbf{16}}_{3}$ of $\mathrm{SO}(10)$.

If some of these bulk states remain in the light spectrum, they could contribute to new physics phenomena with possible signatures in future experiments. We will comment on these issues in the next section.

## Superpotential with $Z_2$ monodromy

Having determined the particle spectrum of the effective field theory model, we proceed now to the superpotential. We find it convenient to perform the analysis using the spectral cover approach. Given that there are two non-trivial $\mathcal{E}_6$ representations available, namely $\mathbf{27}$ and $\mathbf{78}$, the only possible tree-level terms are $\mathbf{27}^3$, $\mathbf{78}^3$ and $\mathbf{78}\cdot \mathbf{78}\cdot \mathbf{1}_x$ where $\mathbf{1}_x$ is a singlet embedded in the $\mathcal{E}_8$ adjoint. We have explained in the introductory section that in the context of the $\mathrm{SU}(3)$ spectral cover the fundamental representation is characterised by the corresponding weights $t_i$ and the Yukawa couplings should respect the requirement $t_1+t_2+t_3=0$. Furthermore, as is well known, a monodromy action is required to ensure a top Yukawa coupling at the tree level. We choose a $Z_2$ monodromy, which identifies the two weights $t_1=t_2$. We accommodate the fermion families in $27_{t_{1}}$ and the Higgs in $27_{t_3}$, so that a diagonal Yukawa term $27_{t_1}27_{t_1}27_{t_3}$ is allowed. After the implementation of the $Z_2$ monodromy, the condition for the weights $t_i$ becomes $2t_1+t_3=0$. It is also worth observing that the spectral cover symmetry reduces essentially to a $U(1)_q$ symmetry in the effective field theory model where the $U(1)_q$ charges of the two matter curves are $t_1$ and $t_3=-2t_1$. Therefore, the symmetry of the effective model is in fact

$$\begin{aligned} \mathrm{SO}(10)\times U(1)_Z\times U(1)_q\subset \mathcal{E}_6\times U(1)_q. \end{aligned}$$

To define the homological properties of the matter curves, we recall that in the case of $\mathrm{SU}(3)_{\perp }$ the spectral cover is described by a cubic polynomial whose roots the $t_i$. For the case of $Z_2$ monodromy we assume the factorisation^5^

15 $$\begin{aligned} b_0s^3+b_2s+b_3 = (a_1+a_2s+a_3s^2) (a_4+a_5 s), \end{aligned}$$

where the $b_k$’s homologies are $[b_k]=\eta -kc_1$ and $[s]=c_1$. Here $\eta =6c_1-t$, where $c_1=c_1(S)$ is the first Chern class of the GUT “surface” *S* and, $c_1(N^{\perp })=-t$ that of the normal bundle.

The second degree polynomial of the right part of the above equation means that two roots are not separable within the field of holomorphic functions, and as a result, a $Z_2$ monodromy identifies the two weights $t_{1}=t_2$ in accordance with our assumptions stated above. Moreover, Eq. (15) implies the following relations $b_k=b_k(a_i)$ between the coefficients:

16 $$\begin{aligned} b_0= & {} a_3a_5, \quad b_1=a_2a_5+a_3a_4=0,\quad b_2=a_1a_5+a_2a_4,\quad \nonumber \\ b_3= & {} a_1a_4, \end{aligned}$$

which can be used to determine the homologies of $a_i$’s. Furhermore, the equation $b_3=a_1a_4=0$ of the $\mathbf{27}$, implies that the two matter curves $27_{t_1}$ and $27_{t_3}$ are associated with the defining equations $a_1=0$ and $a_4=0$ respectively.

From (16), we infer that the homologies satisfy relations of the form $[b_k]=[a_l]+[a_{8-l-k}]$ so that it can be readily found [34, 35] that the $27_{t_1}$ and $27_{t_3}$ homologies are $\eta -2c_1-\chi$ and $\chi -c_1$ respectively where $\chi$ is left unspecified. Then, assuming a *U*(1) flux piercing these matter curves, the multiplicities of $27_{t_i}-\overline{27}_{t_i}$ are given by the restrictions $n_1=\mathcal{F}_{U(1)}\cdot (\eta -2c_1-\chi )$ and $n_3=\mathcal{F}_{U(1)}\cdot (\chi -c_1)$ (with $\mathcal{F}_{U(1)}$ denoting the abelian flux). From this, we deduce that the chiral states of the model are given by

$$\begin{aligned} n_1+n_3 = \mathcal{F}_{U(1)}\cdot (\eta -3c_1)\equiv \mathcal{F}_{U(1)}\cdot (3c_1-t), \end{aligned}$$

and therefore, the unknown homology $\chi$ does not play any rôle in the determination of the chiral spectrum. Hence, to obtain three chiral families we impose $n_1+n_3= \mathcal{F}_{U(1)}\cdot (3c_1-t)=3$ (The flux restrictions, the homologies and other properties of matter curves are shown in Table 1).

Having defined the basic ingredients of the effective model and before we proceed with the implications, a few comments are necessary. We first recall that all the matter fields are effectively representations of the $$\mathrm{SU}(5)\times U(1)_Z$$ gauge symmetry, with $$ U(1)_Z$$ being the linear combination (9) of the two *U*(1)’s embedded in $$\mathcal{E}_8$$. All matter and Higgs fields in the present case transform under the fundamental representation of $$\mathrm{SU}(3)_{\perp }$$. In the spectral cover the latter is replaced by the Cartan subalgebra characterised by the weights $$t_i$$ and hence all GUT fields will be distinguished by an index $$t_i$$.^6^

In the following we will discriminate matter curves with respect to $\mathrm{SU}(3)_{\perp }$ using the subscripts $t_i$ and, to avoid clutter in the formalism, we omit the hypercharge subscript.

The $\mathcal{E}_6$ Yukawa coupling $\mathbf{27}^3$ implies the following generic form of $\mathrm{SO}(10)$ invariants:

17 $$\begin{aligned} \mathcal{W}_{\mathrm{SO}(10)}= & {} \mathbf{16}_{t_i}{} \mathbf{16}_{t_j}{} \mathbf{10}_{t_k}+\mathbf{10}_{t_i}{} \mathbf{10}_{t_j}{} \mathbf{1}_{t_k}, \end{aligned}$$

with *i*, *j*, *k* such that $t_i+t_j+t_k=0$. Under further breaking of the symmetry down to $\mathrm{SU}(5)\times U(1)$, the couplings as well as the associated mass matrices are as follows. The first coupling in (17) implies^7^

18 $$\begin{aligned} \mathbf{16}_{t_i}{} \mathbf{16}_{t_j}{} \mathbf{10}_{t_k}= & {} \left\{ \begin{array}{ll} 10_{t_i}^f10_{t_j}^f5_{t_k}^{h_d}&{}\rightarrow m_{down}\\ 10_{t_i}^f\bar{5}_{t_j}^{h_u}\bar{5}_{t_k}^f&{}\rightarrow m_{up},m_{\nu } \end{array}\right. , \end{aligned}$$

while the second coupling in (17) gives

19 $$\begin{aligned} \mathbf{10}_{t_i}{} \mathbf{10}_{t_j}{} \mathbf{1}_{t_k}= & {} { 5}^{h_d}_{t_i}\,{\bar{5}}^f_{t_j}\,\mathbf{1}_{t_k}+ { 5}^{h_d}_{t_j}\,{\bar{5}}^f_{t_i}\,\mathbf{1}_{t_k}\rightarrow m_{\ell }. \end{aligned}$$

As is expected in flipped models, we observe that the up-quark masses emerge from the $\mathrm{SU}(5)$ coupling $10\cdot \bar{5}\cdot \bar{5}$ and this implies that $\lambda _t=\lambda _{\nu }$ at the GUT scale. The bottom quark masses are obtained from $10\cdot 10\cdot 5$, while the charged lepton masses arise from $\bar{5}\cdot 5\cdot 1$. As a consequence, the well-known relation $\lambda _{\tau }=\lambda _{b}$ at $M_{\mathrm{GUT}}$ of standard $\mathrm{SU}(5)$ is no longer applicable.

The above $\mathrm{SO}(10)$ couplings in general involve potentially dangerous terms leading to baryon and lepton number violation at unacceptable rates. An additional effect of the *U*(1) fluxes introduced to break the symmetry, however, is to ‘eliminate’ various components of the decomposed representations and generate chiralities. Keeping this in mind as well as the required massless spectrum of the effective model, we find that the massless spectrum should be arranged as follows. All families should be accommodated in the $27_{t_{1}}$ curve,

20 $$\begin{aligned} \mathbf{27}_{t_{1}}\rightarrow & {} \mathbf{16}+\mathbf{10}+\mathbf{1}\;\rightarrow \; \{10^f+\phi \}_{t_{1}}+\{\bar{5}^f\}_{t_{1}}+\mathbf{1}_{t_{1}}.\nonumber \\ \end{aligned}$$

In the last step we assume that the flux induces chirality only for the $\mathrm{SU}(5)$ components $10^f$ and $\phi$ of the $\mathbf{16}$-plet and only for $\bar{5}^f$ of the $\mathbf{10}$-plet. On the contrary, the $\bar{5}^{h_u}\in \mathbf{16}$ and $5^{h_d}\in \mathbf{10}$ of $\mathrm{SO}(10)$ are not included, meaning that in the effective theory either they are eliminated or they appear in vector-like pairs with their corresponding (conjugate) representations from the other matter curve.

In a similar way for the Higgses we require

21 $$\begin{aligned} \mathbf{27}_{t_{3}}\rightarrow & {} \mathbf{16}_{t_3}+\mathbf{10}_{t_3}+\mathbf{1}_{t_3}\; \rightarrow \; \{\bar{5}^{h_u}+\phi \}_{t_{3}}+\{ 5^{h_d}\}_{t_{3}}+\mathbf{1}^f_{t_{3}}.\nonumber \\ \end{aligned}$$

In the second step we assume that along the $t_3$ curve, the flux eliminates $10_{t_3}\in \mathbf{16}$ and $\bar{5}^f_{t_3}\in \mathbf{10}$. With this assignment, we readily obtain the following $\mathrm{SU}(5)$ invariant couplings:^8^

22 $$\begin{aligned} \mathcal{W}= & {} 10^f_{t_1} 10^f_{t_2} 5^{h_d}_{t_3} + 10^f_{t_1}\bar{5}^{f}_{t_2}\bar{5}^{h_u}_{t_3} +\bar{5}^{f}_{t_1}1^f_{t_2} 5^{h_d}_{t_3}, \end{aligned}$$

which are just the mass terms for bottom, top, and Dirac neutrino and charged leptons, respectively. To obtain a light Majorana sector we may appeal to the well-known see-saw mechanism which, in F-theory constructions can give an $\mathcal{O}(10^{-1}\mathrm{eV})$ scale from integrating out Kaluza–Klein modes [36].

We emphasise that in our framework the chiral states emerge only from the $\Sigma _{27}$ matter curves. As we have seen in the previous section the chirality of the spectrum is ensured by suitable restrictions of *U*(1) fluxes inside $\mathrm{SU}(3)_{\perp }$. We assume that states coming from the 78 always appear in vector-like pairs. In Sect. 4 we will see how this can be realised, despite the appearance of non-trivial fluxes which will be turned on along *U*(1) factors inside $E_6$.

A possible way to eliminate the unwanted representations can be illustrated in the following simple example. Suppose that on a suitable line bundle, choosing the topological properties as already explained above, we can obtain three chiral $\mathcal{E}_6$ fundamental representations, $n_{27}-n_{\overline{27}}=3$. Turning on an abelian flux along $U(1)_{X'}$ we obtain the following decompositions:

23 $$\begin{aligned} \mathbf{27}_{t_{1}}= & {} \left\{ \begin{array}{ll}\mathrm{Rep.}&{}\;\;\;\#\\ \mathbf{16}_{t_1}&{}:\;x_1\\ \mathbf{10}_{t_1}&{}:\;x_1-n_1\\ \mathbf{1}_{t_1}(e^c)&{}:\;x_1+n_1 \end{array}\right. ,\quad \mathbf{27}_{t_{3}} = \left\{ \begin{array}{ll}\mathrm{Rep.}&{}\;\;\;\#\\ \mathbf{16}_{t_3}&{}:\;x_3\\ \mathbf{10}_{t_3}&{}:\;x_3-n_3\\ \mathbf{1}_{t_3}(\bar{e}^c)&{}:\;x_3+n_3 \end{array}\right. .\nonumber \\ \end{aligned}$$

The integers $$x_{1,3}$$ represent the number of $$\mathbf{27}_{t_1},\mathbf{27}_{t_3}$$ representations and $$n_{1,3}$$ are the integers associated with the flux restrictions on the corresponding matter curves. We wish to accommodate the fermion generations along $$t_1$$ and for the simplest choice $$x_1=3, x_3=0$$ for example, we guarantee the existence of three chiral $$16_{t_1}$$ representations which contain an equal number of $$10\in \mathrm{SU}(5)$$. Furthermore, for the flux we impose the restriction $$\sum _in_i=n_3+n_1=0$$. If we set $$0\le n_1\le x_1$$, we get $$3-n_1\ge 0$$ representations of $$\mathbf{10}_{t_1}\in \mathrm{SO}(10)$$ and $$3+n_1$$ singlets with the quantum numbers of $$e^c$$. On the $$t_3$$ matter curve there are also $$n_1$$ singlet fields transforming in the conjugate representation of $$e^c$$. Hence, three of the $$ 1_{t_1}(e^c)$$ singlets accommodate the $$e^c, \mu ^c, \tau ^c$$, while the remaining $$n_1$$ form vector-like pairs $$(1_{t_1},1_{-t_3})\rightarrow ( E^c,\bar{E}^c)$$. On the contrary, for $$n_1=-1,-2,-3$$ there are only three singlets $$ 1_{t_i}(e^c)$$ available which exactly match the SM states $$e^c, \mu ^c, \tau ^c$$. However, this is no longer true for the 10-plets, and, as can be readily observed, there now exist additional vector-like pairs.

We should point out that if the geometric singularity associated with the GUT symmetry is $\mathrm{SO}(10)$ or higher [28], there is no way to eliminate all the exotic states from the spectrum of the effective model. Therefore, in this $\mathcal{E}_6$ construction additional states always appear in the massless spectrum. Their implications for gauge coupling unification as well as in the effective field theory models emerging from $\mathcal{E}_6$ group have been studied in [35]. The rather interesting fact is that they appear in vector-like pairs so that they can form massive states provided there exist appropriate couplings to singlet fields acquiring vevs.

The number of SU(5) irreps in $$\mathbf{16}$$’s and $$\mathbf{10}$$’s of $$\mathrm{SO}(10)$$ can be similarly defined by the flux parameters along the $$U(1)_X $$:

24 $$\begin{aligned} \mathbf{16}_{t_{i}}= & {} \left\{ \begin{array}{ll} 10^f_{t_i}&{}:\;x_i\\ \bar{5}^{h_u}_{t_1}&{}:\;x_i+m_i\\ 1_{t_i}(\phi )&{}:\;x_i-m_i \end{array}\right. ,\quad \mathbf{10}_{t_{i}}= \left\{ \begin{array}{ll} 5^{h_d}_{t_i}&{}:\;x_i-n_i\\ \bar{5}^f_{t_i}&{} :\;x_i-n_i+p_i\\ \end{array}\right. ,\nonumber \\ \end{aligned}$$

for $i=1,3$ and the total $U(1)_X$- flux is taken to be zero. The flux vanishes automatically on the $16_{t_i}$ components, ($\sum _im_i+(- m_i)=0$), which implies $p_1+p_3=0$.

The values already chosen for $x_{1,3}$ ensure that we have $3\times (10_{t_1}+e^c_{t_1})$ multiplets. However, to obtain three complete families we need $3\times \bar{5}^f_{t_i}$ multiplets. In the simplest scenario we can obtain them from the $t_1$ matter curve, and so we can simply require $x_1-n_1+p_1=3$ which is satisfied for $p_1=n_1$ and $p_3=n_3=-n_1$.

To allow for tree-level invariant Yukawa couplings the Higgs fields are accommodated in $$5^{h_{d}}\in \mathbf{10}_{t_3}$$ and $$\bar{5}^{h_{u}}\in \mathbf{16}_{t_3}$$. In general, depending on the specific choice of the parameters $$n_i, m_i$$, there can be additional vector-like pairs of $$ 5^{h_{d}}_{t_1}, 5^{h_{d}}_{t_3}$$ and $$\bar{5}^{h_{u}}_{t_1}, \bar{5}^{h_{u}}_{t_3}$$ 5-lets but several of them can receive masses from terms of the form $$5_{-t_i}\cdot \bar{5}_{t_j}\cdot 1_{t_i-t_j}$$ or other available couplings.

Next we focus on the $\mathrm{SU}(5)$ breaking. In the case of flipped $\mathrm{SU}(5)$ there exists, in principle, more than one mechanism available for the spontaneous breaking of $\mathrm{SU}(5)$. This can happen either with the hypercharge flux, or with the Higgs mechanism. The hypercharge flux breaking of $\mathrm{SU}(5)$ down to SM splits the $\mathrm{SU}(5)$ multiplets when its restriction on the corresponding matter curve is non-trivial. In general, we wish to maintain intact the representations accommodating the fermions, and hence we may assume that the matter curve $t_1$ is not affected. For the case of Higgs 5-plets, this differentiates the number of doublets and triplets residing on the same matter curve.

We note, however, that there are strong constraints on the spectrum due to the fact that the hypercharge flux should be globally trivial [37–39]. In the present model, however, we can evade such constraints if we appeal to the ordinary Higgs mechanism for the $\mathrm{SU}(5)$ breaking. Indeed, since we deal with flipped $\mathrm{SU}(5)$ [40], we can use the $10_H+\overline{10}_{\bar{H}}$ Higgs-pair to break the symmetry [41]. In this case we assume that the flux also vanishes along the Higgs and matter curves. Such vector-like pairs are then available, and to ensure that these are massless, we require [29] topological properties such that $h^0(\Sigma , K^{1/2}_{\Sigma })=0$, where $\Sigma$ is the corresponding matter curve and $K_{\Sigma }$ the canonical bundle.

For the implementation of this mechanism we will consider a variant of the minimal spectrum presented in Table 4. Assuming the existence of one massless vector-like pair $10^H_{t_1}+\overline{10}^H_{-t_1}$ with content denoted as $10^H=(Q_H,\nu _H^c, D^c_H)$ and $\overline{10}^H=(\bar{Q}_H,\bar{\nu }_H^c, \bar{D}^c_H )$, the following couplings are generated:25As the neutral singlets $\nu _H^c ,\bar{\nu }_H^c$ acquire vevs, the associated triplet pairs become massive and only the corresponding doublet fields $h_u, h_d$ survive ($Q_H, \bar{Q}_H$ are the longitudinal components of gauge bosons). Clearly, this doublet-triplet splitting mechanism affects only one pair of Higgs 5-plets, leaving all others intact. Thus, we end up with the MSSM spectrum and only vector-like 5-plets.

Finally let us comment on two important issues, namely the $\mu$-term and gauge coupling unification. As we have seen, the up- and down-Higgs doublets are accommodated in $\bar{5}_{-2}\in \mathbf{16}_{1}$ and $5_{2}\in \mathbf{10}_{-2}$ representations of $\mathrm{SU}(5)\times U(1)_Z\in \mathrm{SO}(10)\times U(1)_{X'}$. As can be observed, the $U(1)_Z$ symmetry alone, could not solve the naturalness problem, since it would allow a term $\bar{5}_{-2}5_{2}$ with a mass of the order of the GUT scale. Notice, however, that both Higgs 5-plets emerge from the $\mathbf{27}_{t_3}$-matter curve, and therefore this term does not explicitly appear in the Lagrangian because of a non-zero $U(1)_q$ charge (equal to $2t_3$ units), which cannot be cancelled by a tree-level term containing the available neutral singlets capable of developing vevs. Therefore, we can safely appeal to the Giudice–Masiero [42] F-term mechanism for a natural solution of the $\mu$-problem. For the same reason, it is not possible to have a direct tree-level coupling for the colour triplet–antitriplet pair of the Higgs 5-plets, thus avoiding the rapid proton-decay problem.

Next, we will provide a brief account of gauge coupling unification which, in F-theory constructions, is a rather complicated issue. We have seen in the present $E_6$ construction that the massless spectrum contains the MSSM fields, four complete $5+\bar{5}$ pairs and $E^c+\bar{E}^c$ pairs with the quantum numbers of the right-handed electron. The $5+\bar{5}$ pairs modify only the common value of the gauge coupling at $M_{\mathrm{GUT}}$ but do not affect gauge coupling unification. This no longer holds because of the additional $E^c+\bar{E}^c$ fields, but in F-constructions this is not the whole picture.

Indeed, we first point out that the massive Kaluza–Klein modes affect gauge coupling unification since their mass scale (associated also with the right-handed neutrino mass) is comparable to the GUT scale [36]. Moreover, in F-theory derived GUT models there are many massive states charged under the Standard Model gauge group which split the values of the gauge couplings at the GUT scale. Hence, in general we do not expect precise unification [43, 44]. Furthermore, the $E_6$ gauge symmetry breaking via fluxes is another source of gauge couplings’ splitting [45, 46]. In certain cases, it can be shown that several combinations of additional matter can compensate for this splittings leading to a consistent unification scenario. Finally, to keep the hypercharge interaction within the perturbative regime below the GUT scale, some of the extra pairs of 5-plets and $E^c+E$ singlets should receive masses well above the TeV region. This can be achieved if suitable singlet fields acquire appropriate non-zero vevs. A possible way to provide large mass to these fields is by a fourth order non-renormalizable (NR) term of the form $(\theta _{13}+\theta _{1j}\theta _{j3}) \rangle \langle 1_{t_1}\rangle 5_{t_3}^{h_d}\bar{5}_{t_3}^{h_u},$ where either the $\theta _{13}$ singlet or the combination $\theta _{1j}\theta _{j3}, j=4,5$ acquire non-zero vev. A complete account of these issues, however, is beyond the goals of the present work.

## Di-photon emission

As pointed out above, a particularly interesting fact in these F-theory based constructions is the presence of exotic matter in the low energy effective theory. Since matter in these models has a geometric origin, the precise massless content depends on the specific choice of the compactification manifold and the fluxes are parametrised in terms of a few integer parameters.

**Table 2:** $\mathrm{SO}(10)$ origin and multiplicities of the $\mathrm{SU}(5)$ content. If for some choice of the integers $m_1, n_1$ the multiplicity turns negative, then one obtains the conjugate representation. In the last row we include the $\mathcal{E}_6$ singlets $\theta _{13}$ and $\theta _{31}$ with (unspecified) multiplicity *n*

$\mathrm{SO}(10)$	$\mathrm{SU}(5)$	$\#$	$\mathrm{SU}(5)$	$\#$
$\mathbf{16}$	$10_{t_1}^f$	3	$10_{t_3}^f$	0
$\mathbf{10}$	$\bar{5}_{t_1}^f$	3	$\bar{5}_{t_3}^f$	0
$\mathbf{1}$	$1_{t_1}^f(e^c)$	$3+n_1$	$1_{t_3}^f(\bar{e}^c)$	$-n_1$
$\mathbf{10}$	$5_{t_1}^{h_d}$	$3-n_1$	$5_{t_3}^{h_d}$	$n_1$
$\mathbf{16}$	$\bar{5}_{t_1}^{h_u}$	$3+m_1$	$\bar{5}_{t_3}^{h_u}$	$-m_1$
$\mathbf{16}$	$1_{t_1}$	$3-m_1$	$1_{t_3}$	$m_1$
$\mathbf{1}$	$1_{t_1-t_3}$	*n*	$1_{t_3-t_1}$	*n*

**Fig. 1:**
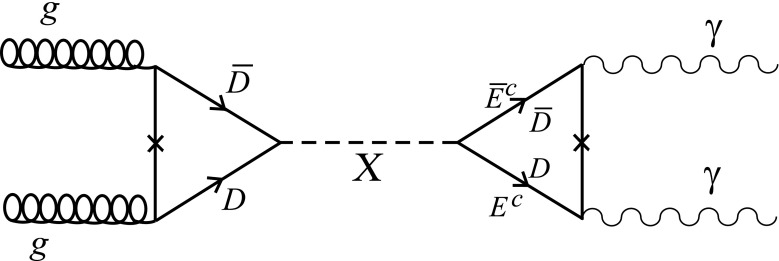
Vector-like representations $E^c+\overline{E^c}$ and $(D+\bar{D})\in (5+\bar{5})$ of $\mathrm{SU}(5)$ contribute to the di-photon signal. The 750 GeV resonance is associated with the MSSM singlets of the model

In the present example this dependence is shown in Table 2. Two specific choices lead to the spectra given below. Starting with a minimal case, we choose $n_1=-m_1=3$ and the resulting spectrum is shown in Table 3. There are three additional vector-like charged leptons with the quantum numbers of $E^c+\bar{E}^c$ which can contribute to the decay of the singlet scalar to two photons as shown in the right loop of Fig. 1.

However, this minimal choice of parameters does not provide vector-like pairs of coloured particles to mediate the *X*-production from gluon fusion, because the couplings of these states to an appropriate singlet are suppressed. This can be achieved if we instead choose $n_1=-m_1=4$. The resulting spectrum is shown in Table 4. There are now four vector-like pairs of $E^c+\bar{E}^c$ as well as the additional pairs $5^{h_d}_{t_3}+ \bar{5}^{\bar{h}_d}_{-t_1}$ and $\bar{5}^{\bar{h}_u}_{-t_1}+\bar{5}^{h_u}_{t_3}$. As a result, they generate the superpotential terms

26 $$\begin{aligned} \bar{5}^{\bar{h}_d}_{-t_1}\cdot 5^{h_d}_{t_3} \cdot \theta _{13}+ 5^{\bar{h}_u}_{-t_1}\cdot \bar{5}^{h_u}_{t_3} \cdot \theta _{13} + (\bar{E}^c)_{-t_3}(E^c)_{t_1}\cdot \theta _{31} .\nonumber \\ \end{aligned}$$

**Table 3:** $\mathrm{SO}(10)$ origin and multiplicities of the $\mathrm{SU}(5)$ content in the first example (see text). The $U(1)_{X}, U(1)_{X'}$ charges of the $\mathrm{SU}(5)$ representations are also shown

$\mathrm{SO}(10)$	$\mathrm{SU}(5)$	$U(1)_{(X,X')}$	$\#$	$\mathrm{SU}(5)$	$U(1)_{(X,X')}$	$\#$
$\mathbf{16}$	$10_{t_1}^f$	$(-1,1)$	3	$10_{t_3}^f$	–	0
$\mathbf{10}$	$\bar{5}_{t_1}^f$	$(-2,-2)$	3	$\bar{5}_{t_3}^f$	–	0
$\mathbf{1}$	$1_{t_1}^f(e^c)$	(0, 4)	6	$1_{-t_3}^f(\bar{e}^c)$	$(0,-4)$	3
$\mathbf{10}$	$5_{t_1}^{h_d}$	–	0	$5_{t_3}^{h_d}$	$(2,-2)$	3
$\mathbf{16}$	$\bar{5}_{t_1}^{h_u}$	–	0	$\bar{5}_{t_3}^{h_u}$	(3, 1)	3
$\mathbf{16}$	$1_{t_1}$	$(-5,1)$	6	$1_{-t_3}$	$(5,-1)$	3
$\mathbf{1}$	$\theta _{13}$	(0, 0)	*n*	$\theta _{31}$	(0, 0)	*n*

We identify the 750 GeV resonance *X* with the singlet $\theta _{13}$ which has the appopriate couplings to give rise to the di-photon diagram shown in Fig. 1. Note that a mixing term $\theta _{13}\theta _{31}$ would allow an additional channel for the resonance to decay into di-photons through the last coupling in Eq. (26). After supersymmetry breaking in the presence of the scalar *X*, the effective Lagrangian contains the terms

27 $$\begin{aligned} \mathcal{L}= & {} \lambda _D\bar{D}DX+\lambda _E\bar{E}^cE^cX+\frac{1}{2}M_X^2XX+A_DX\tilde{D}^*\tilde{D}\nonumber \\&+\, A_EX\tilde{E}^{c*}\tilde{E}^c+\cdots . \end{aligned}$$

We assume here that the singlet field receives a soft mass $M_X$ of the order of the SUSY breaking scale, $\lambda _{f}, f=D,E$ are Yukawa couplings of order one, and $A_{D,E}$ are the trilinear scalar parameters. For a pseudoscalar interaction we should replace the Yukawa coupling according to $\lambda _f\rightarrow i\gamma _5 \lambda _{5f}$.

**Table 4:** The $\mathrm{SU}(5)$ representations, their $U(1)_{X}, U(1)_{X'}$ charges and the corresponding multiplicities for the second case (see text)

$\mathrm{SO}(10)$	$\mathrm{SU}(5)$	$U(1)_{(X,X')}$	$\#$	$\mathrm{SU}(5)$	$U(1)_{(X,X')}$	$\#$
$\mathbf{16}$	$10_{t_1}^f$	$(-1,1)$	3	$10_{t_3}^f$	–	0
$\mathbf{10}$	$\bar{5}_{t_1}^f$	$(-2,-2)$	3	$\bar{5}_{t_3}^f$	–	0
$\mathbf{1}$	$1_{t_1}^f(e^c)$	(0, 4)	7	$1_{-t_3}^f(\bar{e}^c)$	$(0,-4)$	4
$\mathbf{10}$	$\bar{5}_{-t_1}^{\bar{h}_d}$	$(-2,2)$	1	$5_{t_3}^{h_d}$	$(2,-2)$	4
$\mathbf{16}$	$5_{-t_1}^{\bar{h}_u}$	$(-3,-1)$	1	$\bar{5}_{t_3}^{h_u}$	(3, 1)	4
$\mathbf{16}$	$1_{t_1}$	$(-5,1)$	7	$1_{-t_3}$	$(5,-1)$	4
$\mathbf{1}$	$\theta _{13}$	(0, 0)	*n*	$\theta _{31}$	(0, 0)	*n*

Next we provide an estimate of the contributions of the above exotics to the di-photon excess. We assume that the production mechanism of the scalar resonance is mainly from gluon fusion, mediated by loops of the colour triplets, while its decay is mediated by triplets and ($E^c, \bar{E}^c$)-pairs as shown in the figure. The cross section for the scalar mediated process is

$$\begin{aligned}&\sigma (gg\rightarrow X\rightarrow \gamma \gamma )\\&\quad =\frac{1}{M_X\cdot \Gamma \cdot s}C_{gg}\Gamma (X\rightarrow gg)\Gamma (X\rightarrow \gamma \gamma ), \end{aligned}$$

where $\Gamma , \sqrt{s}$ are the total width and the center of mass energy ($\sqrt{s}=13$TeV), respectively, and $C_{gg}$ is the parton integral [47]

$$\begin{aligned} C_{gg}=\int _{M_X/s}^1 f_g(x)f_g\left( \frac{{\tiny M_X}}{sx}\right) \frac{\mathrm{d}x}{x}, \end{aligned}$$

**Fig. 2:**
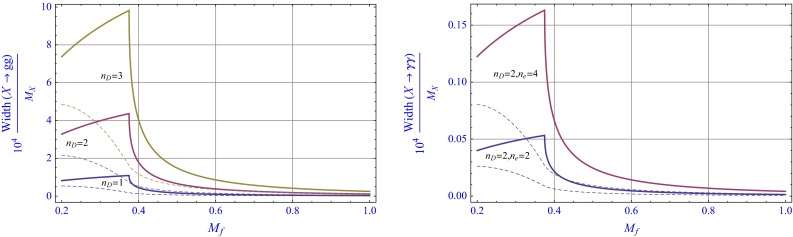
Plots of the ratios $\frac{\Gamma _{gg}}{M_X}$ and $\frac{\Gamma _{\gamma \gamma }}{M_X}$ as a function of the masses of the fermion pairs circulating in the loops of Fig. 1

where $f_g(x)$ is the function representing the gluon distribution inside the proton. The integral is computed using MSTW2008NNLO [47] and its numerical value at 13 TeV is estimated [3] to be $C_{gg}=2137$. The partial widths $\Gamma (X\rightarrow gg),\Gamma (X\rightarrow \gamma \gamma )$ from loops involving fermions and scalars are given by [48] (see also [3])

28 $$\begin{aligned}&\frac{\Gamma (X\rightarrow gg)}{M_X}\nonumber \\&\quad =\frac{\alpha _3^2}{2\pi ^3} \left| \sum _fC_{r_f}\sqrt{\tau _f}\lambda _fS(\tau _f) +\sum _s C_{r_s}\frac{A_s}{2M_X} P(\tau _s)\right| ^2,\nonumber \\ \end{aligned}$$

29 $$\begin{aligned}&\frac{\Gamma (X\rightarrow \gamma \gamma )}{M_X}\nonumber \\&\quad =\frac{\alpha ^2}{16\pi ^3} \left| \sum _fd_{r_f}q_f^2\sqrt{\tau _f}\lambda _fS(\tau _f) +\sum _s d_{r_s}q_s^2\frac{A_s}{2M_X} P(\tau _s)\right| ^2,\nonumber \\ \end{aligned}$$

where $C_r$ is the Dynkin index of the colour representation $(C_r=3$ for the triplet), $d_r$ is its dimension, $q_s$ the charge and $\sqrt{\tau _{a}}=\frac{2 m_{a}}{M_X}$, with $a=f,s$ for the fermion and scalar masses, respectively. The functions $S(\tau ), P(\tau )$ are

$$\begin{aligned} S(\tau ) = 1+(1-\tau ) f(\tau ),\quad P(\tau )=\tau f(\tau )-1, \end{aligned}$$

where [49]

30 $$\begin{aligned} f(\tau )= & {} \left\{ \begin{array}{ll} \arctan ^2\frac{1}{\sqrt{\tau -1}}&{}\quad \tau >1 \\ -\frac{1}{4}\left( \log \frac{1+\sqrt{1-\tau }}{1-\sqrt{1-\tau }}-i\pi \right) ^2;&{}\quad \tau \le 1 \end{array} \right. . \end{aligned}$$

For the pseudoscalar contribution, in the above formulae we make the replacements $S(\tau _f)\rightarrow f(\tau _f), P(\tau _s)\rightarrow 0$ and $\lambda _f \rightarrow \lambda _{5f}$ [3, 48].

For a numerical application, we first consider the existence of only one singlet field *X* with mass $M_X=750$ GeV and, for the sake of simplicity, we take a common mass for the various fermion pairs contributing in the loops. Since the scalar components are expected to be much heavier than the fermions, at this level of approximation their contributions are ignored. In Fig. 2 we plot the widths $\Gamma _{gg}$ and $\Gamma _{\gamma \gamma }$ as a function of the mass of the fermion pairs for two sets of fermion multiplicities for the scalar as well as the pseudoscalar case. If we ignore the large width suggested by the ATLAS data, we observe that there are regions of the fermion mass range where $\Gamma _{gg}\sim \,\mathrm{few}\; 10^{-4}M_X$ and $\Gamma _{\gamma \gamma }\sim \, \mathrm{few}\; 10^{-6}M_X$, which are sufficient to interpret the data. We note in passing that a large decay width allows the exciting possibility of other decay channels including dark matter. In general, however, we expect more than one singlet field with approximately degenerate masses, so that the ATLAS large width could be explained as an unresolved resonance. Another possibility is to invoke additional couplings in the superpotential such as $XH_uH_d$ which permit the resonance to decay into Higgsinos, if kinematically possible, or SM Higgs via the soft trilinear terms.

### Bulk matter

Before closing, we would like to make a final comment on the possible existence of additional ‘exotic’ matter interactions. As we have pointed out, exotic matter arises from the decomposition $\mathbf{78}\rightarrow \mathbf{45}_0+\mathbf{16}_{-3}+\mathbf{\overline{16}}_3+\mathbf{1}_0$ with respect to $\mathrm{SO}(10)\times U(1)_{X'}$ (the indices now refer to $U(1)_{X'}$). We recall that in the twisted model the SM states are in $\mathbf{16}_{-1}$ while bulk states are the $\mathbf{16}_{-3}$, and as such they have exotic charges. Such states could pick up masses at a high scale, in the case that some of them remain light. Due to their large *Y*-hypercharge, they can in principle make a significant contribution to the production and decay of the resonance. As can be observed, all these states come in vector-like pairs, and therefore a possible coupling that could make them massive is

31 $$\begin{aligned} \mathbf{78}\cdot \mathbf{78}\cdot \mathbf{1} \rightarrow M_{Q'} Q'\bar{Q}' +\cdots . \end{aligned}$$

Since these states carry non-zero ‘charges’ under the three *U*(1)’s, in principle, non-trivial fluxes might lead to additional chiral states. Nevertheless, a solution to this problem is feasible if certain topological properties are assumed.

Indeed, we first recall that the number of states is given by the Euler character $\chi$. If $\tau ^*$ is the dual representation of $\tau$, $\mathcal{T}$ is the bundle transforming in the representation *T*, the net number of chiral minus anti-chiral states is given in terms of the formula [50],

$$\begin{aligned} n_{\tau }-n_{\tau ^*}=\chi (S,\mathcal{T}_j^*)-\chi (S,\mathcal{T}_j), \end{aligned}$$

where we assume *S* to be a del Pezzo surface associated with the gauge group $G_S$. If we designate with $\mathcal{L}_j$ a line bundle over *S*, the Euler character is

32 $$\begin{aligned} \chi (S,\mathcal{L}_j)= & {} 1+\frac{1}{2} c_1(\mathcal{L}_j)\cdot c_1(\mathcal{L}_j)+\frac{1}{2} c_1(\mathcal{L}_j)\cdot c_1(S)\nonumber \\ \chi (S,\mathcal{L}_j^*)= & {} 1+\frac{1}{2} c_1(\mathcal{L}_j)\cdot c_1(\mathcal{L}_j)-\frac{1}{2} c_1(\mathcal{L}_j)\cdot c_1(S),\nonumber \\ \end{aligned}$$

so that the difference counting the number of chiral states is

33 $$\begin{aligned} \chi (S,\mathcal{L}_j^*)-\chi (S,\mathcal{L}_j)=- c_1(\mathcal{L}_j)\cdot c_1(S). \end{aligned}$$

We can ensure the vector-like nature of the corresponding states by simply demanding

34 $$\begin{aligned} c_1(\mathcal{L}_j)\cdot c_1(S)=0 \end{aligned}$$

for the particular line bundle.

Focusing now on the $E_6$ case, recall that under the successive breaking we have considered

$$\begin{aligned} E_6&\supset&\mathrm{SO}(10)\times U(1)_X\supset \mathrm{SU}(5)\times U(1)_{X}\times U(1)_{X'}\\&\supset&\mathrm{SU}(3)\times \mathrm{SU}(2)\times U(1)_Y\times U(1)_{X}\times U(1)_{X'}, \end{aligned}$$

while the quantum numbers of the bulk states are

$$\begin{aligned} 78\rightarrow & {} (1,1)_{(0,0,0)}+\left\{ (1,1)_{(0,0,0)}+(1,1)_{(0,0,0)}\right. \nonumber \\&\left. +\,(8,1)_{(0,0,0)}+(1,3)_{(0,0,0)} +(3,2)_{(-5,0,0)}\right. \nonumber \\&+\,(\bar{3},2)_{(5,0,0)}+(3,2)_{(1,4,0)}+(\bar{3},2)_{(-1,-4,0)}\nonumber \\&+\,(\bar{3},1)_{(-4,4,0)} +(3,1)_{(4,-4,0)}+(1,1)_{(6,4,0)}\\&\left. +\,(1,1)_{(-6,-4,0)}\right\} + \left\{ (1,1)_{(0,-5,-3)}+(\bar{3},1)_{(2,3,-3)}\right. \nonumber \\&+\,(1,2)_{(-3,3,-3)} +(1,1)_{(6,-1,-3)} \\&\left. +\,(3,2)_{(1,-1,-3)}+(\bar{3},1)_{(-4,-1,-3)}\right\} \nonumber \\&+\, \left\{ (1,1)_{(0,5,3)}+(3,1)_{(-2,-3,3)}+(1,2)_{(3,-3,3)} \right. \\&\left. +\,(1,1)_{(-6,1,3)}+(\bar{3},2)_{(-1,1,3)}+(3,1)_{(4,1,3)}\right\} . \end{aligned}$$

We can express all the exotics obtained from the decomposition of the $E_6$-adjoint $\mathbf{78}$ in terms of the following three line bundles:

35 $$\begin{aligned} \mathcal{{L}}_1= & {} (5,0,0), \quad \mathcal{{L}}_2 = (1,4,0), \quad { \mathcal {L}}_3 = (1,-1,-3) .\nonumber \\ \end{aligned}$$

It can be shown that by imposing relations analogous to (34) for the three line bundles, all exotic states appear in vector-like pairs and hence, no chiral matter arises from the bulk modes. Moreover, in the minimal case the extra states emerging from $\mathbf{78}$ can assemble in a $5_{ex}+\bar{5}_{ex}$ pair,

$$\begin{aligned} \bar{5}_{ex}= & {} (\bar{3},1)_{(2,3,-3)}+(1,2)_{(-3,3,-3)},\nonumber \\ 5_{ex}= & {} (3,1)_{(-2,-3,3)}+(1,2)_{(3,-3,3)}. \end{aligned}$$

As already stated, these can receive a large mass from terms such as (31), so that gauge coupling unification is not affected.

**Note Added**:

After this paper was submitted for publication the ATLAS and CMS experiments have reported results based on updated analysis including data collected during 2016. This data does not support the presence of the 750 GeV resonance previously reported in 2015 and in Moriond 2016. We should emphasise that our string inspired $E_6$ model predicts the existence of the di-photon resonance as well as vector-like fields. Hopefully, some of these states can be discovered at the LHC and future colliders.

## Conclusions

In this work we have constructed a flipped $\mathrm{SO}(10)\times U(1)$ model fully embedded in an $E_6$ GUT symmetry within an F-theory context. We introduced abelian fluxes along *U*(1)’s inside $\mathcal{E}_6$ to realise the symmetry breaking and generate the chiral families in the low energy spectrum of the effective theory. We have presented simple cases that contain the three chiral families of quarks and leptons. Furthermore, motivated by the 750 GeV di-photon resonance reported by the ATLAS and CMS experiments, we have given examples where the low energy spectrum consists of vector-like fields with a variety of MSSM quantum numbers containing both coloured and leptonic states, as well as gauge singlets. The flipped SO(10) model yields several vector-like $(E^c, \bar{E}^c)$-pairs whose presence could enhance the di-photon decay mode of the scalar resonance.

## Footnotes

1 Although the present experimental data are not sufficient to unambiguously confirm the existence of the resonance event, the discovery of similar resonances at some higher energy scale accessible by the LHC is plausible. In the present model we can, in principle, adjust the free parameters to accommodate such resonances at higher mass scales.

2 There is a vast list of recent publications on possible interpretations of the $\gamma \gamma$ excess. For a comprehensive list of models and interpretations see [27].

3 For previous work on flipped $\mathrm{SO}(10)$ see, for example, [33] and the references therein.

4 For the $U(1)_{X',X}$ charge assignment, we adopt the convention $\mathbf{16}_1\rightarrow 10_{(-1,1)}+\bar{5}_{(3,1)}+1_{(-5,1)}$, $\mathbf{10}_{-2}\rightarrow 5_{(2,-2)}+\bar{5}_{(-2,-2)}$ and $1_{(0,4)}$.

5 Note that, because the sum of the roots is zero, $b_1\equiv \sum _it_i=0$.

6 This is in contrast to $\mathrm{SU}(5)\times \mathrm{SU}(5)_{\perp }$ where, while the $\mathrm{SU}(5)$ GUT 10-plets transform under $5\in \mathrm{SU}(5)_{\perp }$ and carry indices $10_{t_i}$, the GUT 5-plets transform as $10\in \mathrm{SU}(5)_{\perp }$ and therefore are denoted as $\bar{5}_{t_i+t_j}$.

7 Bold face is used for the $\mathrm{SO}(10)$ representations, to avoid confusion with those of $\mathrm{SU}(5)$.

8 In terms of the $U(1)_{X,X'}$ initial charges these couplings are written as follows

$$\begin{aligned} 10^f_{(-1,1)} 10^f_{(-1,1)} 5^{h_d}_{(2,-2)}, \, 10^f_{(-1,1)} \bar{5}^f_{(-2,-2)} 5^{h_u}_{(3,1)},\,10^f_{(-2,-2)} \bar{5}^{h_d}_{(2,-2)} 1^{f}_{(0,4)}. \end{aligned}$$
